# Heat waves impair foraging initiation and directional movement toward a floral scent in the buff-tailed bumblebee (*Bombus terrestris*)

**DOI:** 10.1038/s41598-026-53492-6

**Published:** 2026-07-08

**Authors:** Zoltán Tóth, Brigitta Juhász, Zsolt Kárpáti, Patrick Schultheiss, Sabine S. Nooten

**Affiliations:** 1 Department of Zoology, Plant Protection Institute, HUN-REN Centre for Agricultural Research, Budapest, Hungary; 2 Department of Chemical Ecology, Plant Protection Institute, HUN-REN Centre for Agricultural Research, Budapest, Hungary; 3 Department of Behavioral Physiology and Sociobiology, Biocenter, University of Würzburg, Würzburg, Germany; 4 Department of Animal Ecology and Tropical Biology, Biocenter, University of Würzburg, Würzburg, Germany

**Keywords:** *Bombus*, Flower scent, Wind tunnel, Temperature, Climate change, Experiment, Foraging behaviour, Ecophysiology, Ecology, Physiology

## Abstract

Global climate change is disrupting key ecological processes and species interactions. In particular, the frequency and severity of heat waves have increased dramatically over the last decade. Bumblebees are key pollinators in natural and agricultural systems, representing great economic and biodiversity value. However, these insects are particularly vulnerable to heat stress, because they are exceptionally well adapted to cold environments. Previous studies showed that heat stress has negatively impacted bumblebee foraging in terms of flight performance and foraging success, but its effect on chemosensory orientation is still unknown. In this study, we experimentally investigated if heat wave-treated bumblebees have difficulties in sensing or locating the source of a synthetic floral blend. We found that the proportion of individuals that initiated foraging was significantly lower in the heat wave-treated group than in the control group. Moreover, heat wave-treated bees started foraging later and approached scent sources randomly, although they reached the first scent source with a latency comparable to that of control individuals. Contrary to our initial expectations, the heat wave treatment influenced the antennal response of bumblebees only in a body size-dependent manner. Our findings provide evidence that heat waves can reduce foraging activity and impair directional movements toward floral scents in buff-tailed bumblebees, and support the idea that climate change may be one of the most harmful anthropogenic factors affecting the foraging performance of this pollinator species.

**Supplementary Information:**

The online version contains supplementary material available at 10.1038/s41598-026-53492-6.

## Introduction

Climate change is one of the most significant threats to pollinators, and manifests itself in rising average temperatures as well as increasing frequency and intensity of extreme weather events over the past 30 years^1,2^. In consequence, species shift their distributions leading to alterations in community composition and mismatches in plant-pollinator interactions^3,4^. High temperatures further impact colonies through increased mortality of larvae and adults^5–7^. This can result in further declines of pollinator populations and abundances, with far-reaching consequences for ecological services and ecosystem functioning^8,9^. While there is substantial variation among pollinator species in heat resistance^10–13^, extreme weather events such as heat waves and droughts nevertheless pose a major risk to pollinator-plant interactions in all ecosystems due to their diverse effects on multiple trophic levels^14–17^.

Flowering plants attract pollinators through both visual and olfactory traits^18,19^, and the impact of changes in precipitation and temperature on such traits has been studied extensively^1,20,21^. However, the direct effects of extreme climate events on different aspects of pollinating insects’ foraging behaviour have received less attention^22^. Elevated temperature can disrupt nervous system processes related to perception, increase maintenance energy costs and limit the activity time of foragers, compromising the achievement of optimal energy budgets^23^. In bumblebees, heat wave-level temperatures were found to severely impair foraging-associated learning capabilities and memory^24^, and their occurrence during development weakened the behavioural responses of workers to novel and familiar sensory stimuli^25^. Flight endurance and the likelihood of flight were also shown to be sensitive to thermal conditions, with peak performance at around 25 °C^26^, and heat waves directly reduced the duration of foraging bouts, the proportion of successful bouts and flower visitation through thermal stress on bees^27^. On the other hand, Sepúlveda-Rodríguez et al.^28^ demonstrated that flight speed and the number of workers involved in foraging increased with temperature, and bees did not avoid foraging even at 32 °C despite its potential negative effect on performance. Recent work also found that short breaks may allow foragers to recover from some forms of heat stress^29^, although that may not be true for the olfactory detection of plant volatiles^30^. Thus, the effect of extreme heat events on the chemosensory detection and foraging decisions of pollinating insects represents a pivotal knowledge gap despite the predicted increase in the frequency and intensity of heat waves globally^31^ and the importance of the pollination service these species provide^32^.

The insect olfactory system plays a fundamental role in the perception of and interaction with their environment, with olfactory cues being used extensively to locate mates and suitable host plants for oviposition and food^33–36^. During foraging, pollinating insects track the plumes of volatile floral scents upwind to find potential feeding sites^37,38^, and these chemical cues are considered honest indicators of the amounts of resources provided by the flowering plants^39–41^. This chemical communication between pollinators and plants has been hypothesized to be sensitive to climate change because of the physiological constraints it exerts on both interacting partners^42,43^. It has been shown that temperature variation within the natural thermal range modulates antennal sensitivity to intra- and interspecific chemical cues in various insect species (e.g^44–46^.,).

Such temperature effects on antennal sensitivity are especially concerning if they occur in pollinating insects, potentially disrupting vital pollination services. Bumblebees are key pollinators in natural and agricultural ecosystems of temperate regions^47^, representing great economic and biodiversity value^48–50^. Because of their adaptation to relatively cold climates, these pollinators are predicted to be more severely affected by climate change, as evidenced by the reduction of many *Bombus* species’ distribution in North America and Europe in the last decades^3^. A recent study found that severe heat stress greatly reduced the antennal responses to floral scents in two bumblebee species (*Bombus terrestris* and *B. pascuorum*), without a consistent pattern of recovery 24 h after the heat treatment^30^. Negative impacts on antennal responses were exacerbated when bumblebees were subjected to hot and dry conditions, which constitute the prevailing trend of recent heat waves in Europe^51^.

Here, we build on the results of Nooten et al.^30^ by studying the buff-tailed bumblebee *Bombus terrestris*. We examine the effects of an experimental heat wave (hot and dry conditions) on antennal scent detection and investigate if this impedes the directional movement toward a synthetic floral blend source. We assume that these physiological and behavioural responses are directly linked to foraging performance, i.e., the probability of exploiting a rewarding food source, also in natural circumstances. Based on previous studies^11,26,27,30^, we expect that experimental heat waves would negatively affect bumblebees’ ability to both detect and locate floral scents.

## Methods

### Bumblebee colony management

On 19 June 2024, three super-mini Biobest® hives of *B. terrestris* (each containing more than 40 workers) were acquired from Árpád Biokontroll 2003 Ltd. To minimize potential confounding effects at the colony level on the behavioural measurements, the bees in all three hives originated from the same batch, as per our specific request^52^. The colonies were kept in their original brood boxes, with the top of the cardboard outer box left partially open to enhance ventilation. Ad libitum feeding was provided via an adjacent bottle containing sugar solution (Biogluc®) supplied by the vendor. Beginning five days prior to the initiation of experiments, 20 μL of the undiluted synthetic floral volatile blend (for details, see the ‘Preparation of floral volatiles’ section) was pipetted into the Biogluc® syrup to enable the bumblebees to associate the olfactory cue with a nutritional reward. Additionally, organic flower pollen (ApiLand SRL, Baia Mare, Romania) was supplied to the bees ad libitum. The ambient temperature was 26.8 ± 0.88 °C, and the relative humidity was 61.5 ± 3.75% (measured by two Extech RHT30 (Teledyne Flir LLC, France) data loggers in 30-min intervals). Laboratory illumination was provided exclusively by red light (Philips LED red bulbs; Philips International BV, Amsterdam, The Netherlands) from 04:45 to 21:00 to facilitate bee handling.

### Heat wave treatment

We followed the protocol for simulating the effects of severe weather events (hot and dry) established in Nooten et al.^30^, hereafter termed “heat wave treatment”. Bumblebees assigned to the heat wave treatment group were removed from the colonies 3 h prior to their behavioural trials and placed individually in a 50 ml Falcon tube (Sarstedt, Nümbrecht, Germany) on a foam inlay at 40ºC for 2.75 h. About 10 g of silica gel orange (Roth, Karlsruhe, Germany) was added below the foam to reduce air humidity (mean ± SD: 15 ± 6.2%). The lids were loosely placed on top, to ensure sufficient airflow. The heat treatments were conducted in a climate cabinet (Memmert HPP 110; Memmert GmbH, Schwabach, Germany). After the heat wave treatment, the bees were rested for 15 min to acclimatise to the testing conditions. These parameters were chosen to reflect realistic exposure levels for foraging bumblebees under natural heat wave conditions in Central Europe^51^. They are also known to cause physiological damage to the odour sensing antennae without undue mortality^30^. Control bumblebees were removed from their colonies at the same time as those in the heat wave treatment group and were also kept individually in Falcon tubes and without food for the same amount of time. Controls and resting treated bees were kept under the same conditions as during the behavioural trials (i.e., 26–28 °C and 60–67% RH).

### Preparation of floral volatiles

A synthetic floral volatile mixture was prepared by mixing ten pure synthetic compounds following the methodology of Saunier et al.^53^ and Kárpáti et al.^54^. These individual compounds were combined in specific proportions: 50% benzaldehyde (CAS: 100–52-7), 30% anisaldehyde (CAS: 123-11-5), 8% (Z)-3-hexenyl acetate (CAS: 3681-71-8), 3% β-ocimene (CAS: 13877-91- 3), 2.8% methyl salicylate (CAS: 119-36-8), 2% of (Z)-3-hexen-1-ol (CAS: 928-96-1), 1.4% acetophenone (CAS: 98–86-2), 1.4% benzyl alcohol (CAS: 100-51-6), 1.2% β-caryophyllene (CAS: 87-44-5) and 0.2% phenylacetaldehyde (CAS: 122-78-1). All compounds were purchased from Merck/Sigma-Aldrich, Darmstadt, Germany. The resulting undiluted mixture was added to the Biogluc® syrup prior to the initiation of experiments (see the “Bumblebee colony management” section), and used as one of the scent sources in the behavioural experiment (see in this section below). For the electroantennography (EAG) measurements, we used this undiluted mixture to prepare a dilution series of four different synthetic floral blend concentrations (0.125, 1.25, 12.5, and 125 μg/μl). For that, a filter paper disc (cotton liner, diameter: 12.7 mm; Carl Roth GmbH, Karlsruhe, Germany) was first inserted into a Pasteur pipette. We dissolved the neat synthetic floral blend in mineral oil (CAS: 8042-47-5) to create the above dilutions, then pipetted 10 μl of each dilution directly onto the disc, which served as the stimulus cartridge. For the behavioural experiment, 1 ml of the undiluted synthetic floral blend (same as in^53,54^) and 1 ml of mineral oil as a control were loaded into brown vial-wick dispensers^55^ and used as two scent sources in the behavioural assays.

### Behavioural experiment

**Fig. 1:**
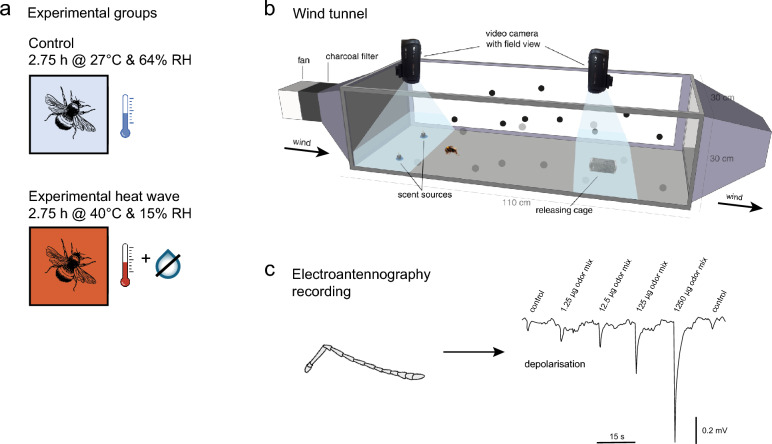
Overview of the experiments to simulate a heat wave and assess the bumblebees’ ability to locate and detect the synthetic floral blend. (**a**) Experimental groups. The control group was exposed for 2.75 h to 27ºC and 62% relative air humidity (RH). The experimental heat wave bees were subjected for 2.75 h to 40ºC and 15% RH. (**b**) Bees were individually transferred to a wind tunnel (110 cm long × 30 cm wide × 30 cm high) with two different scent sources, where their behavioural responses were measured for 10 min. (**c**) The left antenna was then removed from each bumblebee and its antennal response recorded through electroantennography (EAG). The example EAG recording shows the antennal responses of a control group worker bee to the control scent (mineral oil) and the synthetic floral blend of increasing dose (1.25–1250 μg). Panel (**b**) is reproduced with modification from Kárpáti et al.^54^.

Behavioural trials were conducted in a wind tunnel following the protocol of Kárpáti et al.^54^ (Fig. 1). Charcoal-filtered air, maintained at 26–28 °C and 60–67% relative humidity, was propelled through a fine-mesh screen at an airflow rate of 0.1 m s⁻^1^, directed from the scent sources toward the test subjects. An exhaust system located at the distal end of the tunnel vented the wind tunnel air outside the building. Both the synthetic floral blend and the mineral oil were presented via two vial-wick dispensers embedded in the fiberboard base of the wind tunnel; these dispensers were positioned 8.5 cm from the sidewalls and spaced 15 cm apart. To aid orientation toward these scent sources, blue plastic discs (diameter: 2.4 cm) were affixed beneath the black caps of the dispensers as visual cues. Trials started at 9:30 am on each of the four testing days, with 18 bees assigned per day. At the onset of each trial, the scent sources were placed in randomised positions within the wind tunnel, followed by putting the focal individual into an upside down wire mesh cylinder (‘releasing cage’ henceforward; length: 5.5 cm, internal diameter [ID]: 3 cm). The sequence of testing was randomized with respect to colony and treatment. The test subject was allowed to acclimate within the cage for five minutes; the distance between the releasing cage and the scent sources was 65 cm. During this period, illumination was provided exclusively by a red LED light source. After acclimatisation, the observer turned on a white light source simulating daylight (5500–6500 K; Osram Sylvania Luxline Plus light tubes), turned the releasing cage in a lateral position to let the bee leave the cage, and then exited the room. Behaviour of the focal individual at the releasing cage and near the scent sources was recorded from above using two Panasonic HC-V380 video cameras for 10 min. Upon completion, we turned off the white light, so only a red light illuminated the premises again. The bee was then removed from the wind tunnel using a transparent plastic tube (length: 8 cm, ID: 3.3 cm) sealed with a foam insert and set aside for subsequent EAG measurements. After each trial, the releasing cage, dispenser discs, and wind tunnel surfaces were cleaned with 70% ethanol and wiped with paper towels. Continuous airflow between trials ensured the removal of residual synthetic volatiles and alcohol traces from the wind tunnel.

The recorded video files were analysed using the event recorder BORIS v8.25^56^. Each video file was assigned a randomised identification number prior to analysis to avoid observer bias, and the recordings were analysed in a random sequence. From the video data, we extracted the following behavioural parameters: time to leave the releasing cage after the observer turned the releasing cage in a lateral position, time to approach the first scent source, and the type of the first approached scent source. An approach was defined as the event when the focal individual’s head crossed the boundary of the blue disc affixed to the dispenser. In total, we tested 68 bumblebees, out of which 36 were controls and 32 heat wave-treated individuals between 10 and 13th July (four bees died during the heat wave treatment).

### Physiological experiment—electroantennography (EAG)

We performed EAG recordings with the synthetic floral blend to assess the impact of the heat wave treatment on bumblebees’ ability to detect floral volatiles. All bumblebees that participated in the behavioural experiment were tested within 10 min after their trial (*N* = 68). During the EAG experiments, we recorded the electrical response of all olfactory sensory neurons as they react to the synthetic floral blend presented as odour stimuli^57^. For these measurements, a bumblebee antenna was carefully removed and placed inside a glass capillary (internal diameter 1.17 mm, Ockenfels Syntech, Buchenbach, Germany) containing Ringer solution^58^. This capillary was then connected to a silver wire electrode. The half of the last distal antennal segment was cut and inserted into the recording electrode, filled with Ringer solution. The signal was amplified ten times and digitized using an analog–digital converter (IDAC-2, Ockenfels Syntech). For data acquisition, we used GC-EAD software (GC-EAD 2014, version 1.2.5, Ockenfels Syntech).

Each antenna was exposed to odour stimuli for 500 ms, delivered through a continuous flow of humidified, charcoal-filtered air at a rate of 2 l/min, using a Stimulus Controller (CS-55, Ockenfels Syntech). We used mineral oil as the control stimulus, presenting it at both the beginning and end of each experimental session. For each session, four doses of the synthetic blend (1.25, 12.5, 125, and 1250 μg; see the dilution protocol in “Preparation of floral volatiles” section) were tested in ascending order.

### Body size

We measured the length of the marginal cell of bumblebees’ wings after the experiment as a proxy for body size^59,60^. For that, we first cut off each frozen individual’s left forewing from the mesothorax and mounted it between two microscope glass slides with a drop of alcohol to get a completely flattened wing. Then, we photographed the wings using a microscope camera (Oplenic Pro-MicroScan, HangZhou, China) attached to a stereo microscope. We made the photographs compatible with the TPS series^61^ using the program tpsUtil v1.83. Then, we set two landmarks at the proximal and distal end of the marginal cell^62^ on each wing and measured their distance using the program tpsDig2 v2.32.

Additionally, the number of antennal segments was counted for each frozen test subject to verify that the tested individual was a worker. We found that 19 of the 24 bees from the third colony were males, while there were no males in the other two colonies. Because of that, we run the subsequent statistical tests and models on workers (*N* = 49) and males (*N* = 19) separately where applicable (see the “Statistical analyses” section).

### Statistical analyses

All tests were performed in R 4.4.1^63^.. We used Fisher’s Exact Test to compare the proportion of individuals that left the releasing cage in the behavioural experiment between the control and heat wave treatment groups for both workers and males. We could not fit a more complex model on this binary outcome (with the value of 1 if the individual left the cage and 0 if the individual did not leave the cage) due to the separation issue resulting from all control individuals leaving the releasing cage. A mixed-effects Cox proportional hazards model (‘coxme’ R package^64^) was employed to assess how the starting time of trial (decimal time as a covariate), body size (covariate), heat wave treatment (factor), and the interaction between body size and heat wave treatment influenced the latency of workers to exit the releasing cage (if they did so during the behavioural experiment, *N* = 38). We fitted a generalised linear mixed model (GLMM) with binomial error distribution using Template Model Builder (‘glmmTMB’ R Package^65^) to investigate how the starting time of trial, body size, heat wave treatment, and the interaction of the latter two affected the type of the first-approached scent source in workers (with the value of 1 for ‘floral blend’ and 0 for ‘mineral oil’, *N* = 32). A second mixed-effects Cox proportional hazards model was used to evaluate the effects of the starting time of trial, body size, the interaction between the first-approached scent source type and heat wave treatment, and the interaction between body size and heat wave treatment on the time required for workers to approach the first scent source. In these latter two models, only those workers were included that approached at least one scent source during the behavioural trial (*N* = 32). Compliance with the proportional hazard assumption was confirmed for both fitted Cox models. Model assumptions for the generalized linear mixed model (GLMM) were verified through residual diagnostics using the ‘DHARMa’ R package^66^. We included ‘Day’ crossed with ‘Colony’ in these three mixed-effect models as a random term. Similar models were not fitted on males’ data as only two heat wave-treated individuals left the releasing cage and none of them approached any scent source during the behavioural experiment.

To investigate the impact of heat wave treatment on antennal responses to the floral blend at four concentrations in the physiological experiment, GLMMs with Gaussian error distribution were fitted using the ‘glmmTMB’ R package. For these analyses, electroantennogram (EAG) amplitudes were first normalised by averaging the responses to mineral oil control stimuli recorded before and after each session^54^. Subsequently, EAG amplitudes were adjusted by subtracting this averaged control response to compensate for solvent and mechanoreceptive responses^54,67,68^. Data were log-transformed (log(*x* + 0.1)) to improve conformity with normality assumptions. To analyse whether workers’ antennal response differed between nests, we used only the control group (i.e., responses of bumblebees that did not undergo the heat wave treatment) and fitted a model with colony, concentration (both as factors) and their interaction as potential predictors and ‘Individual identity’ and ‘Testing day’ as crossed random effects (*N* = 26). In addition, we used this model to test for a ‘dose–response’ by comparing responses among concentrations. To investigate the effects of heat wave treatments on antennal responses and whether body size plays a role in workers, we fitted the similar model as described above with heat wave treatment (as a factor), body size (as a covariate), concentration (as a factor) and all their interactions (including the three-way interaction) as fixed effects (*N* = 49). The random term included the ‘Individual identity’ nested in ‘Colony identity’, and crossed with ‘Testing day’. Post hoc comparisons were used to assess differences between control and heat wave treatment within each concentration. We also fitted similar models (but without colony as a predictor or a random factor) on males’ data, but due to a convergence problem indicated by the performed residual diagnostics or a significant deviation of residuals from normal distribution in the final model, we do not report the results from these models.

Finally, we investigated potential differences in body size between tested workers from the three colonies using a generalized linear model (GLM; ‘lme4’ R package^69^) with radial cell length as the response variable and colony identity (as a factor), treatment (as a factor), and their interaction as potential predictors.

The significance of fixed effects in all models was determined using type II Wald χ^2^ tests via the Anova function of the ‘car’ R package^70^. Post hoc comparisons were performed with FDR (for comparisons to a null hypothesis) or Tukey HSD (for pairwise comparisons) adjustment using the ‘emmeans’ R package^71^. We used the ‘emtrends’ function from this package to characterise the antennal response-body size relationship separately in the two treatment groups (averaged over the levels of concentration). All tests were two-tailed with α set to 0.05. The detailed treatment design is given in Table S1 (Supplementary materials).

### Ethical note

No permits or ethical approvals were necessary for conducting the experiment using commercially available buff-tailed bumblebees in Hungary. Upon completion of the study, bees were euthanized by freezing to prevent any potential mixing with the native population.

## Results

### Behavioural experiment

**Fig. 2:**
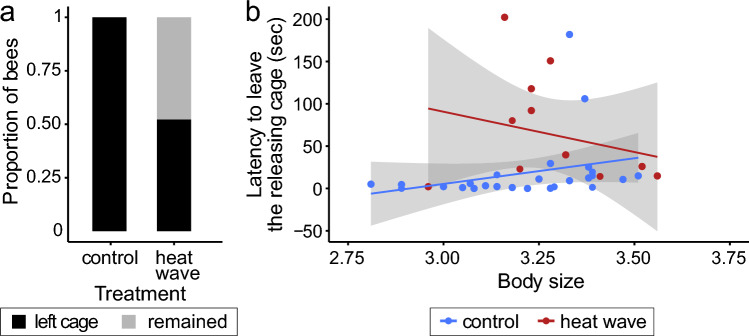
Behavioural responses of *B. terrestris* workers at the beginning of the behavioural experiment. (**a**) The proportion of focal individuals that left the releasing cage in the two treatment groups (black: left the releasing cage; grey: remained in the releasing cage; *N* = 49). (**b**) The relationship between latency of leaving the releasing cage and body size (in mm) in the two treatment groups (blue: control; red: heat wave-treated individuals; *N* = 38). We used the length of the marginal cell of bumblebees’ wings as a proxy for body size. Regression lines are shown with 95% confidence intervals.

**Fig. 3:**
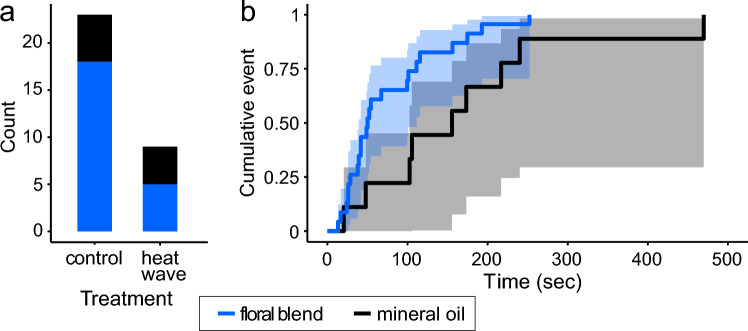
Behavioural responses of workers after leaving the releasing cage to the presented scent sources (*N* = 32). (**a**) The number of focal individuals that approached the blend-containing (blue) and the mineral oil-containing (black) scent sources in the two treatment groups. (**b**) Kaplan–Meier curves for the cumulative incidences of reaching the first scent source (blue: blend-containing scent source; black: mineral oil). Curves are shown with 95% confidence intervals. For graphical presentation only, we used the ‘survfit’ function of the ‘survival’ R package^72,73^ without including the random term.

At the start of the trials, the proportion of workers that left the releasing cage was significantly lower in the heat wave treatment group than in the control (heat wave-treated bees: 12 out of 23; control: 26 out of 26; *P* < 0.001; Fig. 2a). Heat wave-treated workers leaving the cage did so significantly later than their control counterparts, but this effect was also influenced by body size (*χ*^2^_1_ = 3.96, *P* = 0.046; Table 1): latency slightly decreased with body size in the heat-wave treated, but increased in the control individuals (Fig. 2b). The starting time of trial did not affect this measure (Table 1). We found no significant difference in the proportion of workers that chose the blend-containing scent source first between the treatment groups (*χ*^2^_1_ = 1.59, *P* = 0.208), but this might be due to the low sample size in the heat wave treatment group (*N* = 9) resulting in a high uncertainty associated with the treatment effect. In line with that, post hoc comparisons revealed that the estimated probability of approaching the blend-containing scent source first was significantly higher than the 50% expected by chance in the control (estimate ± SE: 0.78 ± 0.09, *z*-ratio = 2.53, *P* = 0.023) but not in the heat wave-treated workers (0.56 ± 0.17, *z*-ratio = 0.33, *P* = 0.739; Fig. 3a). Other investigated predictors, such as starting time of trial or body size, did not affect this measure either (all *P* ≥ 0.446; Table 1). Workers approached the synthetic floral blend significantly sooner than the mineral oil (*χ*^2^_1_ = 6.98, *P* = 0.008; Fig. 3b). The heat wave treatment, however, had no significant effect on this latency either by itself or in interaction with the type of the first-approached scent source (both *P* ≥ 0.738). The time to approach the first scent source decreased marginally significantly as the starting time of trial increased (*χ*^2^_1_ = 3.14, *P* = 0.076). Body size or its interaction with treatment did not influence the latency to approach the first scent source (both *P* ≥ 0.199; Table 1).

**Table 1:** Test statistics and the significance of the explanatory variables from the mixed-effect models fitted on the investigated behavioural parameters in *B. terrestris* workers.

Response variable	Model type	Predictors	*χ*^2^	df	*P*
Latency to leave the releasing cage (*N* = 38)	Mixed-effect Cox model	Starting time of trial	0.31	1	0.581
		Body size	**5.34**	**1**	**0.021**
		Heat wave treatment	**6.41**	**1**	**0.011**
		Heat wave treatment × body size	**3.96**	**1**	**0.046**
Type of the first-approached scent source (*N* = 32)	GLMM with binomial error distribution	Starting time of trial	0.58	1	0.446
		Body size	0.05	1	0.816
		Heat wave treatment	1.59	1	0.208
		Heat wave treatment × body size	0.04	1	0.838
Latency to approach the first scent source (*N* = 32)	Mixed-effect Cox model	Starting time of trial	3.14	1	0.076
		Body size	1.35	1	0.246
		Heat wave treatment	< 0.01	1	0.969
		Type of scent source	**6.98**	**1**	**0.008**
		Heat wave treatment × type of scent source	0.11	1	0.738
		Heat wave treatment × body size	1.65	1	0.199

Significant predictors in the final models and their values are shown in bold. Test statistics and *P*-values for the non-significant predictors were computed by including them one by one in the final models.

The proportion of males that left the releasing cage was also significantly lower in the heat wave treatment group than in the control (heat wave-treated bees: 2 out of 9; control: 10 out of 10; *P* = 0.001). Due to the low sample size of individuals that left the releasing cage in the heat wave treatment group, we did not perform any further analysis in males.

### Physiological experiment—EAG

Antennal responses of control workers to the synthetic floral blend were dose-dependent and significantly increased with concentration in the control colonies (*χ*^2^_3_ = 2215.8, *P* < 0.001; Table 2; Fig. S1). Responses were on average lowest at 1.25 μg and increased by 30-fold at 1250 μg dose. There was no significant difference in antennal response among individuals from the three colonies in general or in interaction with concentration (both *P* ≥ 0.377; Fig. S1).

**Table 2:** Summary of the GLMMs fitted on the antennal responses of workers to the synthetic floral blend.

Response variable	Predictors	*χ*^2^	df	*P*
Antennal response (only the control group; *N* = 26)	Concentration	**2215.8**	**3**	** < 0.001**
	Colony	1.95	2	0.377
	Concentration × colony	2.35	6	0.885
Antennal response (both control and treatment groups; *N* = 49)	Concentration	**3695.09**	**3**	** < 0.001**
	Body size	**6.77**	**1**	**0.009**
	Heat wave treatment	1.95	1	0.162
	Heat wave treatment × body size	**3.89**	**1**	**0.048**
	Concentration × body size	3.46	3	0.326
	Concentration × heat wave treatment	3.15	3	0.369
	Concentration × body size × heat wave treatment	0.58	3	0.901

Significant predictors in the final models and their values are shown in bold. Test statistics and *P*-values for the non-significant predictors were computed by including them one by one in the final models.

**Fig. 4:**
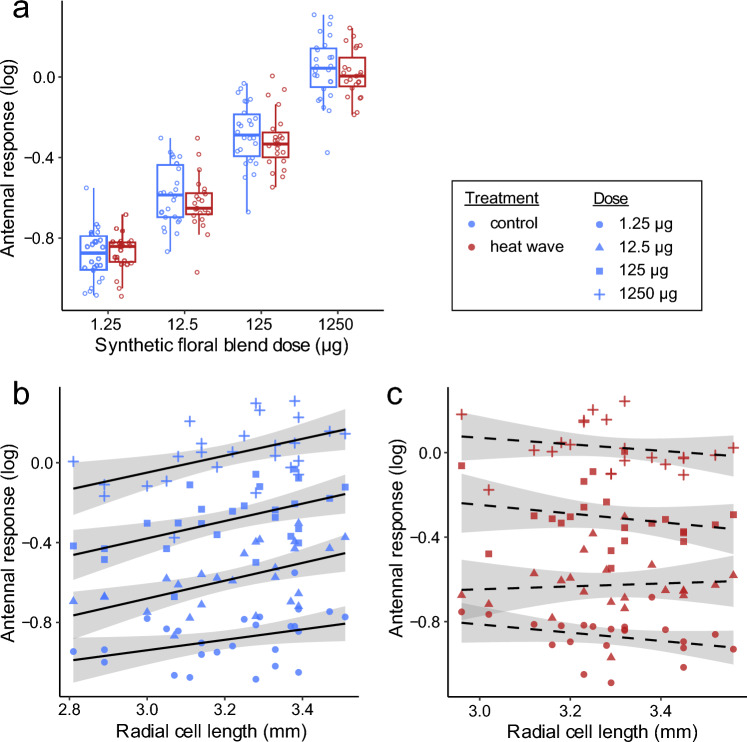
Antennal responses of control and heat wave-treated *B. terrestris* workers to the synthetic floral blend at four different doses (*N* = 49). (**a**) Boxplots show the median, upper and lower quartile. Circles show antennal responses on log scale. Scatter plots show antennal response (on log scale) in relation to body size for (**b**) the control group and (**c**) the experimental heat wave group. Solid black lines imply significant relationships between traits and variables (at *P* < 0.05), dashed black lines show non-significant ones, grey shaded area shows 95% confidence interval.

Antennal responses were also dose-dependent and increased significantly with concentration (*χ*^2^_3_ = 3695.09, *P* < 0.001; Table 2) for both the control and heat wave treatment group. Heat wave treatment had no significant effect on bumblebees’ antennal response in itself, only in interaction with body size (*χ*^2^_1_ = 3.89, *P* = 0.048; Fig. 4a). The observed pattern indicated that antennal responses increased with body size in the control group (estimated trend ± SE: 0.29 ± 0.09, *t*-ratio = 3.21, *P* = 0.002), but this relationship disappeared after the experimental heat wave (0.04 ± 0.09, *t*-ratio = 0.49, *P* = 0.623; Fig. 4b,c).

### Differences in workers’ body size between colonies

Bumblebee body size, measured as marginal cell length, ranged from 2.81 to 3.56 mm. We found no difference in this measure between the three colonies in workers (*χ*^2^_2_ = 0.35, *P* = 0.841; Fig. S2a), and there was no difference in body size between individuals in the control and the experimental heat wave group within colonies either (*χ*^2^_2_ = 0.49, *P* = 0.784; Fig. S2b).

## Discussion

For large pollinating insects like bumblebees, the energy demands of foraging are particularly high due to the cost of flying, necessitating a careful balance of energy intake throughout their foraging trips^74^. However, elevated temperature can disrupt optimal energy budget^s23^. Here, we showed that extreme weather conditions, such as a short period of abnormally high temperature, induce substantial changes in the foraging behaviour of buff-tailed bumblebees. Specifically, we found that only about half of the heat wave-treated workers left the releasing cage in the wind-tunnel tests. Even when they did so, bees started foraging later and did not choose preferentially the floral blend source over the control stimulus, although they reached the first scent source with similar latency to control individuals. Contrary to our initial prediction, the heat wave treatment affected the antennal response of workers only in a body size-dependent manner. Our results provide experimental evidence that exposure to high temperatures can drastically reduce buff-tailed bumblebees’ ability to initiate foraging trips and discriminate between rewarding and unrewarding scent sources. Because of that, frequent heat waves may be especially detrimental to foraging activity and pollination performance, and thus to individual and colony fitness, in this cold-adapted pollinator species.

The main finding of this study was that a substantial proportion of bumblebees, both workers and males, did not engage in foraging-related behaviour following the heat wave treatment. Among the workers that remained active, we detected no significant difference between heat wave-treated bees and controls, but the small sample size of the heat wave-treated bees likely limited statistical power in this analysis. When groups were analysed separately, control bees approached first the synthetic floral blend more frequently than expected under random choice, indicating discrimination of the rewarding scent source, whereas heat wave-treated workers did not deviate from random expectation. This difference suggests impaired directional movement toward floral scents after heat stress (in line with previous findings^26,27^;), and may have remained subtle because the analysed individuals likely represent those least affected by the treatment, as many more strongly affected bees did not initiate foraging behaviour at all. In addition, behavioural assays were conducted after a short recovery period rather than during active heat wave conditions, suggesting that the observed responses reflect carry-over effects of prior heat wave exposure. Differences in response latency further suggest some level of size-dependent participation after the heat wave treatment (Fig. 2b), even though the choice of synthetic floral blend or mineral oil was not dependent on body size (Fig. S4). Bumblebees in natural conditions are able to compensate some of the heat wave effects through behavioural strategies of thermoregulation including seeking out thermal refuges and adjusting their temporal activity pattern^75^, and thus some of our findings potentially indicate variation in heat wave resistance among workers of different body sizes^12,76^. The behavioural effects of heat stress observed in this study are in line with previous findings (e.g^26,27,77^.,) and provide additional evidence for the detrimental consequences of elevated temperature on foraging initiation. We found that many heat wave-treated bumblebees did not show any foraging activity and remained inert, but those individuals that started foraging were similarly likely to approach the blend and control scent sources. However, sensory capability was not reduced in these individuals, as individual antennal and behavioural responses to the floral blend were not correlated (see in Supplementary materials, Fig. S3). The lower proportion of heat wave-treated bees that initiated a foraging bout thus indicates a reduced motivation or ability to search for food, either through disruption of motor control^78^ or a behavioural response aimed at diminishing the adverse effects of heat stress. Many insects, including bumblebees, can regulate their body temperature through phenotypic plasticity^28^. For instance, heat-exposed individuals can actively divert warm haemolymph to well-ventilated body parts, such as the abdomen and potentially the head, to cool down their thorax^79^^80^,. This may take time to implement and come into effect, during which period all other energy-intensive activities are delayed. Even then, foragers’ ability to distinguish between different sources of scents would be essential for effective resource exploitation. This observed inability to differentiate between different scent sources implies heat-induced constraints on cognitive functions such as foraging-associated learning and memory^24^ or behavioural sensitivity to chemical stimuli^25^. It has been hypothesized that high temperature may disrupt neuronal signalling in the periphery sensory systems of pollinating insects and/or in the brain regions that are important for learning and memory, such as the antennal lobe, the mushroom bodies and the central complex^24,81,82^, although the exact mechanism behind this is poorly understood. Nevertheless, heat stress during development has been demonstrated to greatly impair associative odour learning (but not memory) without affecting detection sensitivity through its detrimental effect on the mushroom body in adult *Drosophila melanogaster*^83^.

We showed that, in general, control and heat wave-treated bumblebees had similar antennal responses to the synthetic floral blend. This result seemingly contradicts previous findings of Nooten et al.^30^, who found that heat waves induce strong reductions in antennal responses to floral scents in this species. However, there were important differences in the methods used between the two studies, which may explain, at least partly, the observed discrepancy. First, here we used a blend of 10 floral volatiles as a scent source, whereas Nooten et al.^30^ tested the detection of three floral volatile organic compounds (VOCs) separately; it is known that the detection limits of blends and single compounds can differ substantially^84,85^. Second, we allowed bees to rest for 15 min after the heat wave treatment, then conducted the 10-min-long behavioural experiment in a well-ventilated wind tunnel, and measured their antennal response within a further 10 min. In the study by Nooten et al.^30^, most bumblebees were tested directly after the heat wave treatments, so there was much less time for the treated test subjects to recover. We further found that antennal sensitivity increased with body size in the control bees. This confirms previous findings that the antennae of larger individuals have higher EAG responses than those of smaller bumblebees^86^. We found, however, that this positive relationship between antennal sensitivity and body size diminished in the heat wave treated-individuals. This implies that size-related superior odour detection and foraging efficiency^87,88^ may diminish during heat waves, further limiting the total amount of nectar that workers can gather in these extreme weather conditions. Our result is not attributed to size differences between the treatment groups, as we observed no variation in body size between the treated and control bees. While developmental temperature is known to adversely impact certain morphological traits in bumblebees^6,89,90^, the duration of the heat wave treatment in our study was too brief to produce any noticeable effect on the radial cell length, which we used as a proxy for body size.

It is important to note that our study focused solely on the effects of heat waves on the pollinator, without considering its interaction with the pollinated plant. It has long been established that elevated temperatures can increase floral scent production and volatility (e.g^91^.,), potentially enhancing plant-pollinator interactions^92^; but see^17,93^). In theory, higher emissions of VOCs could offset a decrease in the antennal detection ability of heat wave-exposed foragers. However, we observed no behavioural responses to high concentrations of chemosensory cues (note that we used the neat blend of VOCs in the behavioral experiment) in most heat wave-treated individuals. For that reason, an increased VOC production is unlikely to bolster significantly the foraging activity and orientation ability of bees under similar heat stress. In addition, the present study investigated heat wave effects on pollinator foraging and physiology without considering any other abiotic factors that are associated with ongoing climate change, such as drought spells, elevated CO₂ and/or habitat loss. Examining the interactions of these factors may provide valuable insights into the ecological consequences of climate change. Future studies should also test interactive effects of multiple climate change associated abiotic factors to elicit impacts on pollinator foraging behaviour and physiology.

Our results indicate that the experimental heat wave substantially affected not only workers, but also males, potentially reducing their foraging activity, mating success, or survival. Previous studies suggest that males and workers have similar abilities to learn and utilize floral cues during foraging^94–96^, although males may have less motivation to participate in cognitive assessments offering nutritional rewards^97^. A likely reason for this difference is that workers forage for both themselves and the colony, while males forage only for their own energy demands^98^. Besides, male buff-tailed bumblebees carry less pollen on their bodies and visit fewer flowers per day than workers do. However, previous measurements of pollen loads in males suggest that males may also contribute substantially to pollination services^99^. This potential contribution highlights the importance of understanding how environmental stressors such as heat waves affect males as well.

In summary, we showed that heat waves can substantially influence the foraging behaviour of buff-tailed bumblebees through reduced activity and impaired directional movement toward a floral scent. We provide the first experimental evidence that bees exposed to heat waves may become unable to discriminate between rewarding and control scent sources even if their floral blend detection ability remains largely unaffected. Our findings support the assumption that climate change can be one of the most detrimental anthropogenic influences on the foraging performance of this pollinator species through the observed heat wave-induced behavioural responses.

## Supplementary Information

Supplementary Information.

## Data Availability

Data supporting the results and R scripts for the statistical analyses are archived and available at Figshare (https://figshare.com/s/f5ba6e21dc4bfc1e1c51).
