# Supplementary material for: Heat waves impair foraging initiation and directional movement toward a floral scent in the buff-tailed bumblebee (Bombus terrestris)
Source: Sci Rep. 2026 Jul 8;16:21196. doi: 10.1038/s41598-026-53492-6 (PMC13346528; doi:10.1038/s41598-026-53492-6)
Supplement: Supplementary file 1 — Supplementary Information. [file 41598_2026_53492_MOESM1_ESM.pdf]

Supplementary material

Heat waves impair foraging initiation and directional movement toward a floral scent in the buff-tailed bumblebee (*Bombus terrestris*)

Zoltán Tóth, Brigitta Juhász, Zsolt Kárpáti, Patrick Schultheiss & Sabine S. Nooten

This supplement contains:

Supplementary Figures S1 to S4

Supplementary Table S1

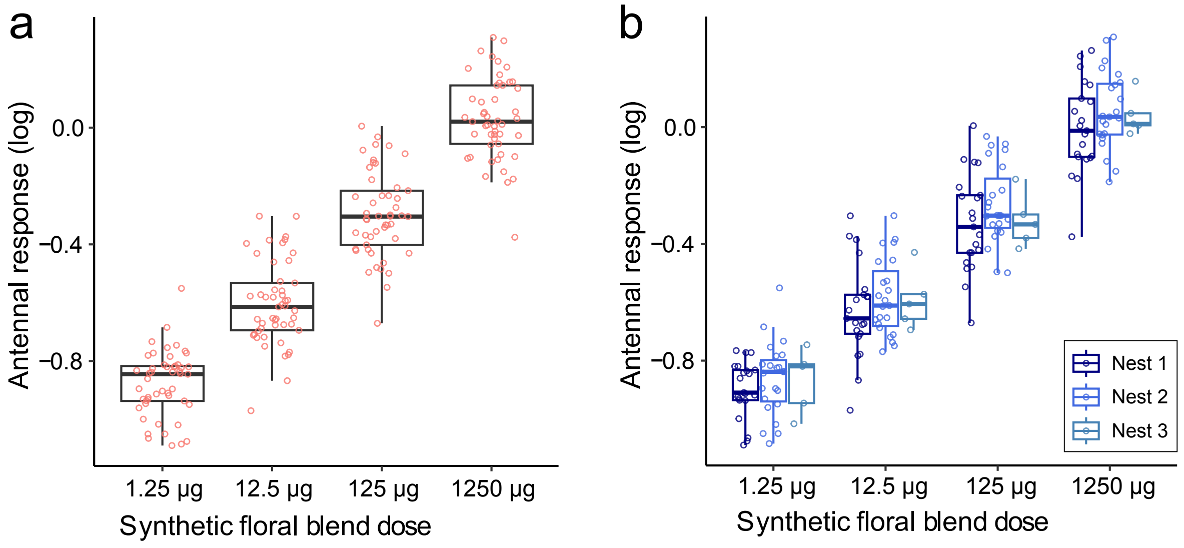

Fig. S1: Antennal responses of *B. terrestris* workers from three colonies to a synthetic floral blend at four concentrations. (a) Overall responses of bees from all nests. (b) Antennal responses of the three different experimental nests. Boxplots show the median, upper and lower quartile. Circles show antennal responses on log scale.

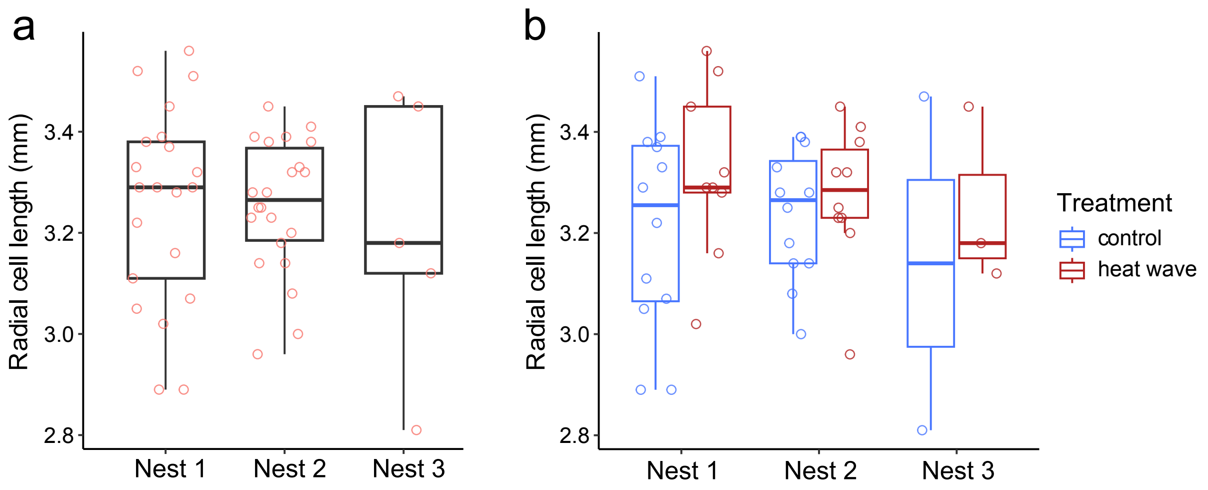

Fig. S2: Body size (measured as radial cell length) of *Bombus terrestris* workers (a) from the three colonies, and (b) for control and heat wave-treated individuals from the three colonies.

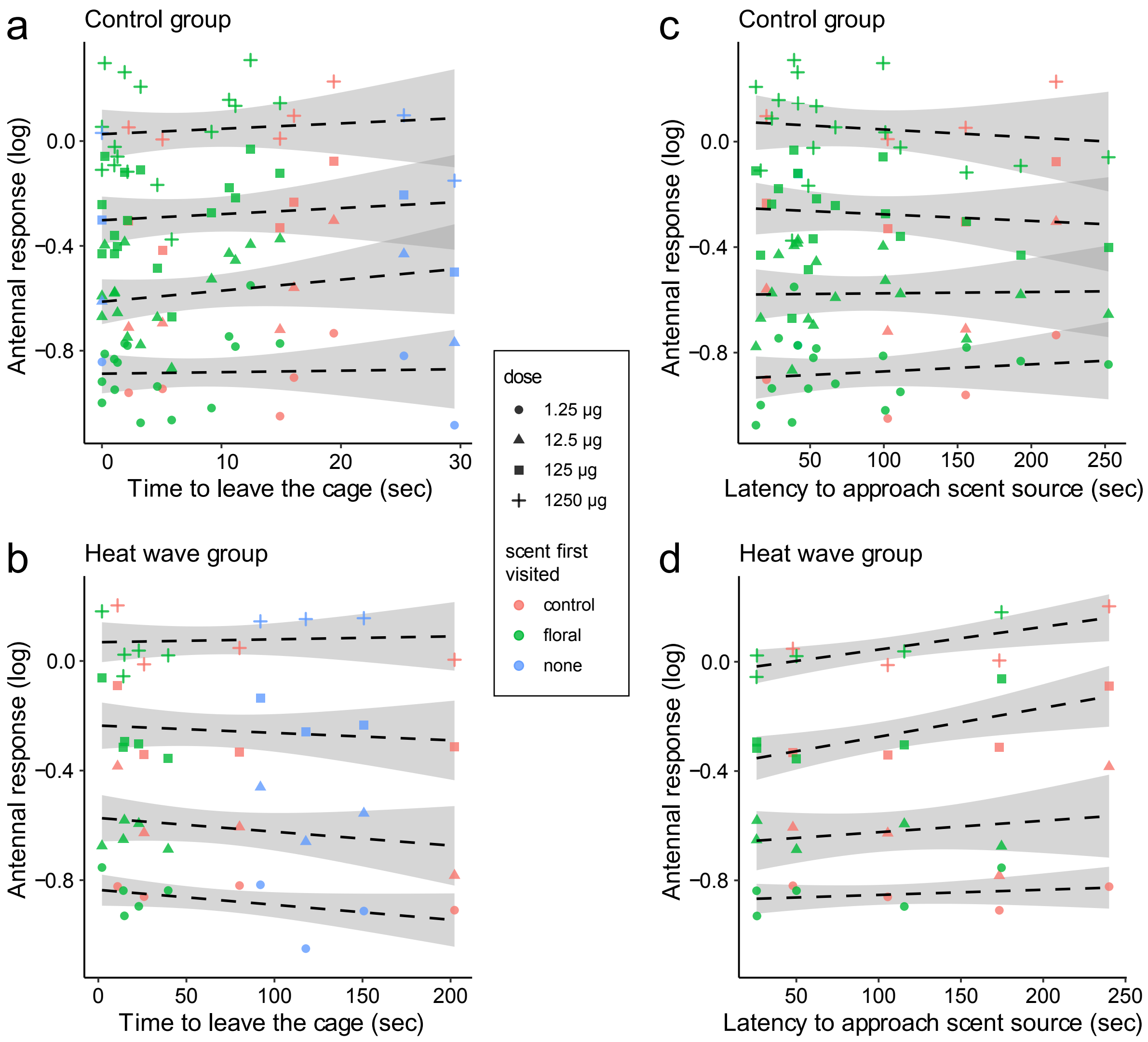

Fig. S3: Relationships between physiological and behavioural responses of *B. terrestris* workers. Left panels show antennal responses (on log scale) and time to leave the releasing cage for (a) the control and (b) the heat wave group; right panels show antennal responses and latency to approach the scent source. Four doses of floral blend are shown in different icons, and colours indicate the scent type: control (red), floral blend (green), and none (blue).

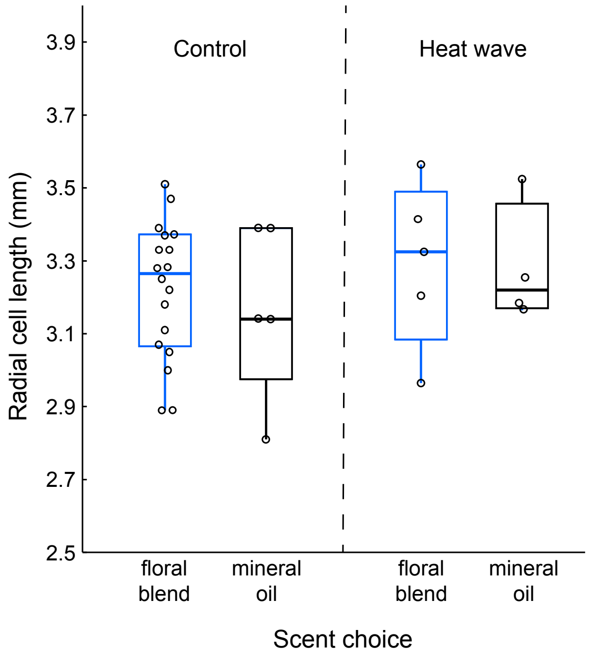

Fig. S4: Body size – measured as length of the radial cell in the left forewing (mm) - of the bumblebees approaching the synthetic floral blend or the mineral oil, for both treatment groups (control, heat wave).

Table S1: Detailed treatment design and sample sizes of bumblebee workers and males for the physiological experiments (EAGs) and the behavioural assays in the wind tunnel (behaviour).

	worker			males
Treatment	Physiology (EAG)	Behaviour (leave cage)	Behaviour (scent approach)	Behaviour (leave cage)
control	26	26 out of 26	23 out of 26	10 out of 10
heat wave	23	12 out of 23	9 out of 12	2 out of 9
sum	*49*	*38*	*32*	*12*
